# Incidence of retinoblastoma in children and adolescents in Brazil: A population-based study

**DOI:** 10.3389/fped.2022.1048792

**Published:** 2022-11-23

**Authors:** Annamaria Ciminelli Barbosa, Maria Clara de Magalhães-Barbosa, Jessica Pronestino de Lima Moreira, Giovanni Nicola Umberto Italiano Colombini, Arnaldo Prata-Barbosa

**Affiliations:** ^1^ Department of Pediatrics, D’Or Institute for Research & Education (IDOR), Rio de Janeiro, RJ, Brazil; ^2^ Department of Ophthalmology, Federal University of the State of Rio de Janeiro, Rio de Janeiro, RJ, Brazil; ^3^ Institute of Public Health Studies (IESC), Federal University of Rio de Janeiro, Rio de Janeiro, RJ, Brazil; ^4^ Instituto de Puericultura e Pediatria Martagão Gesteira, Federal University of Rio de Janeiro, Rio de Janeiro, RJ, Brazil

**Keywords:** retinoblastoma, incidence, cancer, pediatrics, Brazil

## Abstract

**Objective:**

To estimate the incidence of retinoblastoma in children and adolescents in Brazil based on Population-Based Cancer Registry (PBCR), describing temporal trends and some quality indicators of this registry.

**Methods:**

Based on secondary data from the PBCR of the National Institute of Cancer (INCA) (2000–2018), by selecting the morphological code of retinoblastoma, the annual incidences per million (0–19 years of age) in each local PBCR were estimated by sex and age group, global combined and by region, in addition to the percentage of diagnosis only by death certificate (DC) or not informed (NI), and the male/female ratio (M/F). An annual incidence trend in the five Brazilian geographic regions was also analyzed using the inflection point regression technique.

**Results:**

675 patients were identified in 28 PBCR, 91% between 0 and 4 years of age. The overall combined incidence per million by age group was: 7.02 (0–4 years old), ranging from 5.25 in the Midwest to 11.26 in the Northeast; 0.46 (5–9 years old); 0.05 (10–14 years old) and 0.03 (15–19 years old). The combined incidence per million, adjusted for the world population, was 2.23 (0–14 years old) and 2.24 (0–19 years old). The DC and NI percentages were 13% and 18%, respectively; the M/F ratio was 1.3. The incidence remained stable throughout the study period, except for the city of São Paulo, where there was a significant reduction of 3.4% per year.

**Conclusions:**

In Brazil, except for the Northeast region, the incidences of retinoblastoma were lower than those reported in several countries worldwide, suggesting possible underreporting, and the time series analysis showed a stable trend. Although this pioneering study brings a recent panel of available data on retinoblastoma in Brazil, more precise estimates are needed and welcome for better planning of onco-ophthalmologic care in the country.

## Introduction

Retinoblastoma is the most common primary malignant tumor in childhood (1). The molecular cancer mechanism is related to a mutation of the RB1 gene, a tumor suppressor gene located in region 1, band 4, of the long arm of chromosome 13 (13q14). The tumor develops when both alleles of the RB1 gene suffer a loss of function, making cell cycle control unfeasible (2). Mutations are inherited as an autosomal dominant trait and passed on to offspring (3, 4).

The tumor has a higher incidence in the age group of one to four years, and although some studies show a slight male predominance (5–8), other studies do not show a difference between the sexes (9–14). The main form of ocular involvement is unilateral. Its bilaterality is rare and usually associated with cases with a positive family inheritance (1). As retinoblastoma is very aggressive, early diagnosis and prompt treatment are essential for survival.

Although it accounts for about 3% of childhood malignancies in developed countries, there is evidence that this tumor occurs more frequently in Latin America and Africa developing countries (1). Studies show that in the age group from 0 to 14 years, the standardized incidence rate per one million children varies from 2.9 to 4.4 in Asian countries (15–22), and from 3.4 to 6.5 in countries in Europe (13, 14, 23–26), from 3.2 to 4.7 in North America (5, 6, 10–12, 27, 28), from 6.7 in Central America, Guatemala (9), and from 4 to 5 in South American countries (8, 29, 30).

The incidence of retinoblastoma increases with decreasing age. Countries in North America, Europe, and Asia have reported retinoblastoma incidence rates of 10 to 12 per million children under five years of age (5, 10–12, 22–25), while Mexico has reported a rate of 20.8 cases of retinoblastoma per 1 million children under one year of age (6). In this sense, although retinoblastoma is a tumor with a low incidence in general, in children under one year of age, the incidence can reach 20/1,000,000, which generates high morbidity related to the disease.

The assessment of cancer incidence is paramount for effective prevention and control actions. The Cancer Incidence in Five Continents (CIFC) report (31), produced by the International Agency for Research on Cancer (IARC), linked to the World Health Organization, is an essential source of reference in populations throughout the world and is based on data from local Population-Based Cancer Registries (PBCR) in each country. PBCRs store all new cancer cases that occur in a defined population, from a given geographic area, through an ongoing and systematic data collection process (29).

There are currently 31 PBCRs implemented in Brazil, whose organization and management have been under the responsibility of the National Cancer Institute (INCA) since the 1980s (29). INCA uses the IARC recommendations and disseminates them, qualifying the Brazilian registry teams. The latest CIFC Vol. X includes data from only 5 PBCRs from Brazil. In addition, this report has no itemized incidence estimates of retinoblastoma. Despite being a relatively uncommon tumor in the general population, it represents 2 to 4% of neoplasms in children under 15 and 15.4% in children under one year of age. The latest INCA report on cancer incidence in children and adolescents in Brazil was published in 2016 with data from varying periods among the different local PBCRs, going up to 2012 at the latest (7).

In this sense, the knowledge of the most up-to-date incidence rates of retinoblastoma in the different pediatric age groups in Brazilian regions can provide information about the actual magnitude of the problem and bring improvements in planning public health policies related to pediatric oncology. This study aims to present more comprehensive and more up-to-date estimates of retinoblastoma incidence rates in children and adolescents in Brazil, based on Population-Based Cancer Registries, describing temporal trends and some quality indicators of these registries, related to retinoblastoma.

## Materials and methods

### Ethics approval

The study was approved by the Research Ethics Committee of the D'Or Institute for Research and Education (IDOR), under the n° 5,480,625 (June 21, 2022), which waived the need for informed consent.

### Study design and data setting

This is an incidence study, including children and adolescents aged 0 to 19 years diagnosed with retinoblastoma from 2000 to 2018, based on secondary data from the Population-Based Cancer Registries (PBCR), managed by the National Cancer Institute (INCA), linked to the Ministry of Health of Brazil.

### Features of the population-based cancer registry (PBCR)

PBCRs aim to collect, analyze and classify all new cancer cases to produce reliable statistics of these occurrences in a defined population and to provide an organized framework for establishing and controlling cancer's impact on the community in the coverage area of the registry (29). New cases are identified in patients with proven residence in the area covered by the PBCR, with a diagnosis confirmed by anatomopathological examinations, surgical exploration, autopsy, death certificate, or any other means of diagnosis with authorization from the doctor responsible for the patient.

The reporting sources are cancer hospitals, general hospitals, university hospitals, specialized clinics (oncology and terminal patients), medical offices, nursing homes, diagnostic centers (pathological anatomy, clinical analysis and imaging, cancer treatment centers (radiotherapy and chemotherapy), and Health Departments (Health Information Systems). The percentage of cases with histological diagnosis, with diagnosis only by the death certificate, and the ratio between the number of male and female patients (M/F) are some of the quality indicators recommended in the Manual of Routines and Procedures of the PBCR that can be extracted (28). PBCR are the only registries that allow calculating tumor incidences in each population.

### Data collection and statistical analysis

Individualized and unidentified data from cancer patients registered in all PBCR in Brazil, with a diagnosis date from 2000 to 2018, were requested from INCA by filling out a specific form available on its website (http://www.inca.gov.br). Then, the following successive filters were used: 0 to 19 years of age, malignant neoplasm of eyes and appendages (code C69-2 of International Classification of Diseases version 10, ICD-10), malignant neoplasm of the retina (code C69-2, ICD-10), and retinoblastoma morphology without other specifications, differentiated, undifferentiated, and diffuse (International Classification of Diseases for Oncology, 3rd edition, ICD-O-3, codes 9510/3, 9511/3, 9512/3, and 9513/3, respectively). The population estimates used as denominators for the calculation of incidence rates were obtained from DATASUS (https://datasus.saude.gov.br) (32) and the Brazilian Institute of Geography and Statistics (IBGE).

Crude incidence rates (IR) of retinoblastoma (RTB) per one million children and adolescents were calculated as the ratio between the total number of retinoblastoma cases in each local PBCR in a given period and the population at risk in the coverage area of the respective PBCR in the same period.


$$\mathrm{IR}=\frac{\begin{array}{c} \mathrm{Total}\;\mathrm{number}\;\mathrm{of}\;\mathrm{RTB}\;\mathrm{cases}\;\mathrm{in}\;a\;\mathrm{given}\\ \mathrm{PBCR}\;\mathrm{in}\;a\;\mathrm{defined}\;\;\mathrm{period} \end{array}\;}{\begin{array}{c} \mathrm{Population}\;\mathrm{of}\;\mathrm{the}\;\mathrm{coverage}\;\mathrm{area}\;\mathrm{of}\;a\;\mathrm{given}\\ \mathrm{PBCR}\;\mathrm{in}\;a\;\mathrm{defined}\;\;\mathrm{period}\; \end{array}}\times 1,000,000$$

To provide the best estimate of the incidence of retinoblastoma in Brazil, we chose to estimate the combined incidence rate (CIR) using the following formula:

$$\mathrm{CIR}=\frac{\begin{array}{c} \sum \mathrm{Number}\;\mathrm{of}\;\mathrm{RTB}\;\mathrm{cases}\;\mathrm{of}\;\mathrm{all}\;\mathrm{PBCRs}\\ \mathrm{in}\;\mathrm{certain}\;\;\mathrm{periods} \end{array}}{\begin{array}{c} \sum \;\mathrm{population}\;\mathrm{of}\;\mathrm{coverage}\;\mathrm{areas}\;\mathrm{of}\;\mathrm{all}\;\\ \mathrm{PBCRs}\;\mathrm{in}\;\mathrm{the}\;\mathrm{respective}\;\mathrm{periods} \end{array}}\times 1,000,000$$

Incidence rates were stratified by region, sex, and age group (0 to 4 years, 5 to 9 years, 10 to 14 years, 15 to 19 years, and 0 to 19 years). To calculate the stratified incidences, the populations at risk in the denominators were also stratified by region, sex, and age group.

Age-standardized incidence rates (ASIR), for each local PBCR and Brazil, were calculated using the direct method and the standard world population used in the publications of the International Cancer Incidence on Five Continents/IARC series. Such a population was based on a combination of the age structures of developed and developing countries (IARC, 1995).


$$\mathrm{ASIR}=\frac{\sum \left(\mathrm{age}-\mathrm{specific}\;\mathrm{rate}\right)\times \left(\begin{array}{c} \mathrm{standard}\;\mathrm{world}\;\\ \mathrm{population}\;\mathrm{in}\;\mathrm{age}\;\mathrm{group} \end{array}\right)}{\sum \mathrm{Standard}\;\mathrm{world}\;\;\mathrm{population}}\times 1,000,000$$

For the evaluation of temporal trends, local PBCR data from each of the five regions of the country that had long reference periods were used, from the year 2000 to the year 2014, with the cities of Belém/Ananindeua, Recife, Brasília Belo Horizonte, São Paulo, and Curitiba being selected for this purpose. The temporal trend of these cities together was also analyzed. For these calculations, the software Joinpoint Regression Program, version 4.9.0 (Statistical Research and Applications Branch, Surveillance Research Program, National Cancer Institute, USA) was used, which uses the technique of regression by inflection points (“joinpoints”), a segmented linear regression with correction for first-order autocorrelation, in which changes in the correlation between incidence rate (dependent variable) and calendar year (independent variable) are connected by “joinpoints”, whose fluctuation is smoothed by the natural logarithmic transformation of the dependent variable. The various segments generated are explored through Monte Carlo permutation tests to choose the model that best explains the trend over time, starting from a minimum number of inflection points and testing whether adding more “joinpoints” to the model is statistically significant (33). The Annual Percent Change (APC) and their respective 95% confidence intervals (95% CI) were calculated for the generated “joinpoints”, and the final model was selected. To quantify the summary measure of trends over the entire period, the Average Annual Percent Change (AAPC) was also calculated, estimated by the weighted geometric mean of the different APCs with weight equal to the segment size for each time interval. The statistical significance level was set at 0.05. Significant values represent an increasing trend if the value is positive and decreasing trend if negative. Non-significant values represent a stationary (stable) trend.

To assess the quality of the PBCR, some indicators were calculated, according to the recommendation of the Manual of Routines and Procedures for Population-Based Cancer Registries of INCA (29), as described below.

I. Percentage of cases diagnosed only by death certificate (DC):
  $$\text{\%}\;\mathrm{diag}.\;\mathrm{DC}=\frac{\begin{array}{c} \mathrm{No}.\;\mathrm{of}\;\mathrm{retinoblastoma}\;\mathrm{cases}\;\mathrm{as}\;\\ \mathrm{means}\;\mathrm{of}\;\mathrm{DC}\;\mathrm{diagnosis} \end{array}}{\mathrm{Total}\;\mathrm{number}\;\mathrm{of}\;\mathrm{retinoblastoma}\;\mathrm{cases}}$$

II. Percentage of cases with unreported diagnosis:
  $$\text{\%}\;\;\mathrm{diag}.\;\mathrm{hist}=\frac{\begin{array}{c} \mathrm{No}.\;\mathrm{of}\;\mathrm{retinoblastoma}\;\mathrm{cases}\;\mathrm{as}\;\\ a\;\mathrm{means}\;\mathrm{of}\;\mathrm{diagnosis}\;\mathrm{not}\;\mathrm{informed} \end{array}}{\mathrm{Total}\;\mathrm{number}\;\mathrm{of}\;\mathrm{retinoblastoma}\;\mathrm{cases}}$$

III. Male/Female ratio:
  $$M/F\;\mathrm{ratio}\;=\frac{\mathrm{No}.\;\mathrm{of}\;\mathrm{retinoblastoma}\;\mathrm{cases}\;\mathrm{in}\;\mathrm{males}}{\mathrm{No}.\;\mathrm{of}\;\mathrm{retinoblastoma}\;\mathrm{cases}\;\mathrm{in}\;\;\mathrm{females}}$$

## Results

**Figure 1:**
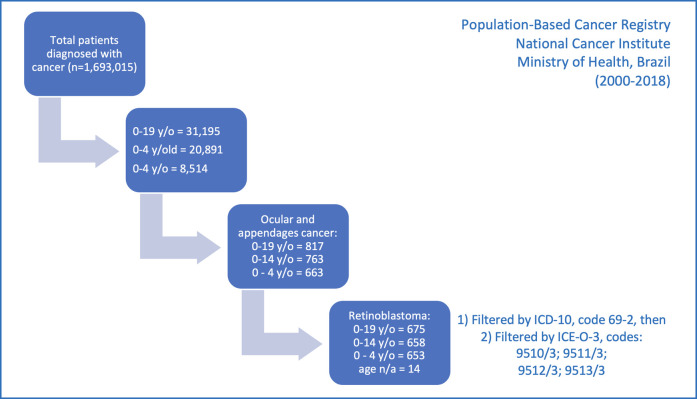
Selection of children and adolescents diagnosed with retinoblastoma, from 2000 to 2018, in Brazil, included in the Population-Based Cancer Registries of the National Cancer Institute, Ministry of Health, Brazil.

**Figure 2:**
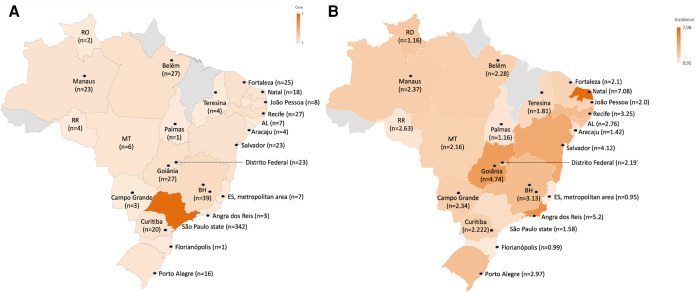
Number of retinoblastoma cases (0–19 years of age) incident in the five Brazilian geographic regions, according to the Population-Based Cancer Registry of the National Cancer Institute, Ministry of Health of Brazil. **Northern Region:** RO (Rondônia state), RR (Roraima state) and cities of Manaus (Amazonas state), Belém and Ananindêua (Pará state) and Palmas (Tocantins state). **Northeast Region:** AL (state of Alagoas) and cities of Fortaleza (state of Ceará), Natal (state of Rio Grande do Norte), João Pessoa (state of Paraíba), Recife (state of Pernambuco), Aracaju (state of Sergipe), Teresina (state of Piauí) and Salvador (state of Bahia). **Midwest Region:** MT (state of Mato Grosso) and cities of Goiânia (state of Goiás), Campo Grande (state of Mato Grosso do Sul) and Brasília (Federal District). **Southeast Region:** cities of São Paulo, Barretos, Campinas and Jahu (state of São Paulo); Belo Horizonte (state of Minas Gerais); Espírito Santo state metropolitan region (cities of Cariacica, Guarapari, Serra, Viana, Vila Velha, Vitória and Fundão); Angra dos Reis (state of Rio de Janeiro). **South Region:** cities of Curitiba (state of Paraná), Florianópolis (state of Santa Catarina) and Porto Alegre (state of Rio Grande do Sul).

A total of 675 children and adolescents aged 0 to 19 years with a diagnosis of retinoblastoma were identified in 28 PBCR from different regions of Brazil from 2000 to 2018, corresponding to 2.2% of all cancer cases in this age group (Figure 1). Of these cases, 55.4% were male, 90.4% were between 0 and 4 years old (representing 7.2% of all cancer cases in this age group), half were white, and the majority (58%) lived in the southeastern region of Brazil (Table 1). The diagnostic method was the histology of the primary tumor in 91.6% of the cases. Still, the description of other clinical characteristics, such as laterality and morphology, was hampered by a large number of missing values (Table 2). The distribution of the absolute number of cases across the five regions of the country is shown in Figure 2A and Supplementary Table S1.

**Table 1:** Demographic characteristics of retinoblastoma cases from the Population-Based Cancer Registry, 2000–2018, National Cancer Institute, Ministry of Health, Brazil.

Features	*n* = 675	%
**Sex**		
Male	374	55.41
Female	289	42.81
**Age group (years)**		
<1	166	24.59
1–4	444	65.78
5–9	41	6.07
10–14	5	0.74
15–19	3	0.44
No information	16	2.37
**Race/Color**		
White	160	50.00
Brown	138	43.13
Black	14	4.38
Yellow	7	2.19
Indigenous	1	0.31
No information	360	
**Origin (place of residence)**		
Southeast	391	57.93
Northeast	116	17.19
Midwest	74	10.96
North	57	8.45
South	37	5.48

**Table 2:** Clinical characteristics of analytical cases of retinoblastoma from the Population-Based Cancer Registry, 2000–2018, National Cancer Institute, Ministry of Health, Brazil.

Features	*n* = 675	%
**Laterality**		
Right	15	34.09
Left	17	38.64
Bilateral	12	27.27
No information	631	
**Morphology**		
Retinoblastoma, NOS	624	92.44
Diffuse Retinoblastoma	1	0.15
Differentiated Retinoblastoma	35	5.19
Undifferentiated Retinoblastoma	10	1.48
Malignant neoplasm	4	0.59
Malignant giant cell tumor	1	0.15
**Diagnostic method**		
Cytology	1	0.15%
Clinical	10	1.50%
Histology of the primary tumor	608	91.57%
Histology of metastasis	1	0.15%
Tumor markers	1	0.15%
Research	39	5.87%
Only the death certificate	4	0.60%
No information	11	

NOS, no other specifications.

The crude incidence of the local RCPBs in the 0–19 years of age group varied from 0.32 (Mato Grosso, inland) to 7.08 (Natal) (Figure 2B and Table 3). The combined crude incidence rates of all local PBCR in Brazil in the age groups 0 to 4, 0 to 14, and 0 to 19 years were respectively 7.02, 2.48, and 1.82 per million (Table 3). In the 0–4 age group, the crude combined incidence rates ranged from 5.25 per million in the Midwest region to 11.26 per million in the Northeast region, where the PBCR in the city of Natal had an incidence rate of 27.9 per million, the highest among all local PBCRs. From 0 to 14 years old and from 0 to 19 years old, the highest combined incidence rates adjusted by the world standard population (ASIR) were registered in the Northeast region (3.73/1,000,000; 0–19 years), followed by the region South (3.04/1,000,000; 0–19 years old), North (2.58/1,000,000; 0–19 years old), Southeast (2.34/1,000,000; 0–19 years old) and Center– west (1.75/1,000,000; 0–19 years) (Table 3). These crude and adjusted incidence rates, distributed according to sex, are shown in Supplementary Table S2.

**Table 3:** Crude and adjusted incidence rates of retinoblastoma per 1 million children and adolescents aged 0 to 19 years, by local Population-Based Cancer Register (PBCR) and respective reference periods, National Cancer Institute, Ministry of Health, Brazil.

Region	PBCR	Age group (years)							
		0–4	5–9	10–14	15–19	0–14		0–19	
		IR	IR	IR	IR	IR	ASIR	IR	ASIR
North	Manaus (2000–2013)	8.67	0.82	0,00	0.00	3.16	2.81	2.37	2.81
	Belém e Ananindêua (2000–2017)	8.45	1.03	0.33	0.00	3.12	2.87	2.28	2.87
	Roraima (2003–2010)	2.54	2.52	0.00	0.00	3.41	1.39	2.63	1.39
	Rondônia (2015–2017)	4.88	0.00	0.00	0.00	1.59	1.47	1.16	1.47
	Palmas (2000–2013)	3.59	0.00	0.00	0.00	1.19	1.08	0.88	1.08
**Combined incidence**		**7.71**	**0** **.** **94**	**0** **.** **15**	**0** **.** **00**	**2** **.** **97**	**2** **.** **58**	**2** **.** **20**	**2** **.** **58**
Northeast	Natal (2000–2008)	27.94	3.28	0.00	0.00	9.81	9.20	7.08	9.20
	Salvador (2000–20005)	16.40	1.51	0.00	0.00	5.81	5.30	4.12	5.30
	Alagoas (2010–2011)	12.10	0.00	0.00	0.00	3.69	3.63	2.76	3.63
	Recife (2000–2016)	11.60	1.01	0.47	0.87	4.16	3.85	3.25	4.05
	João Pessoa (2000–2016)	8.90	0.00	0.00	0.00	2.77	2.67	2.00	2.67
	Fortaleza (2000–2013)	8.22	1.05	0.00	0.00	2.90	2.73	2.10	2.73
	Teresina (2000–2016)	5.84	1.93	0.00	0.00	2.52	2.23	1.81	2.23
	Aracaju (2000–2014)	4.61	1.50	0.00	0.00	1.97	1.76	1.42	1.76
**Combined incidence**		**11.26**	**1** **.** **15**	**0** **.** **10**	**0** **.** **18**	**3** **.** **78**	**3** **.** **69**	**2** **.** **91**	**3** **.** **73**
Midwest	Goiânia (2000–2013)	19.20	0.74	0.69	0.00	6.60	6.10	4.74	6.10
	Campo Grande (2008–2012)	9.98	0.00	0.00	0.00	3.23	2.99	2.34	2.99
	Distrito Federal (2000–2014)	7.96	0.95	0.00	0.00	2.96	2.62	2.19	2.62
	Cuiabá/Várzea Grande (2000–2016)	7.83	1.29	0.00	0.00	2.97	2.67	2.16	2,35
	Mato Grosso (inland) (2001–2016)	0.87	0.42	0.00	0.00	0.42	0.37	0.32	0.37
**Combined incidence**		**5.25**	**0** **.** **63**	**0** **.** **08**	**0** **.** **00**	**4** **.** **29**	**1** **.** **75**	**1** **.** **43**	**1** **.** **75**
Southeast	Angra dos Reis (2007–2016)	22.80	0.00	0.00	0.00	7.10	6.84	5.20	6.84
	Barretos DRS (2000–2018)	15.21	1.84	0.00	0.00	5.46	5.02	3.96	5.02
	Belo Horizonte (2000–2017)	13.55	0.68	0.00	0.00	4.39	4.24	3.13	4.24
	Campinas (2002–2016)	6.40	0.00	0.85	0.00	2.35	2.11	1.70	2.11
	São Paulo (2000–2015)	6.07	0.22	0.00	0.00	2.11	1.88	1.55	1.88
	Metropolitan area, ES* (2000–2012)	3.45	0.00	0.54	0.00	1.30	1.16	0.95	1.16
	Jahu (2000–2018)	5.70	0.00	0.00	0.00	1.86	1.71	1.36	1.71
**Combined incidence**		**7.58**	**0** **.** **24**	**0** **.** **03**	**0** **.** **00**	**4** **.** **96**	**2** **.** **02**	**6** **.** **84**	**2** **.** **34**
South	Porto Alegre (200–2012)	12.27	0.77	0.00	0.00	4.10	3.87	2.97	3.87
	Curitiba (2000–2016)	8.78	0.47	0.00	0.40	2.93	2.75	2.22	2.84
	Florianópolis (2008–2016)	4.49	0.00	0.00	0.00	1.42	1.35	0.99	1.35
**Combined incidence**		**9.53**	**0** **.** **52**	**0** **.** **00**	**0** **.** **22**	**4** **.** **99**	**2** **.** **99**	**2** **.** **36**	**3** **.** **04**
**Total combined incidence**		**7.02**	**0** **.** **46**	**0** **.** **05**	**0** **.** **03**	**2** **.** **48**	**2** **.** **23**	**1** **.** **82**	**2** **.** **24**

ASIR, adjusted incidence rate, for the world standard population; IR, crude incidence rate.

*ES = Espírito Santo state (cities of): Cariacica, Guarapari, Serra, Viana, Vila Velha, Vitória, and Fundão.

In absolute numbers, most retinoblastoma cases were concentrated in the Southeast region (*n* = 391). To assess the percentage of retinoblastoma cases identified only by the death certificate (DC) or not informed, a total of 691 cases were considered, including, in addition to the 675 cases with a morphological diagnosis of retinoblastoma, another 16 cases characterized as malignant neoplasm of the retina, all under seven years of age, due to the high probability of being cases of retinoblastoma as well. Among these, the mean percentage of cases with DC diagnostic was 1.9% (*n* = 13) and with uninformed diagnostic means was 2.6% (*n* = 18). Considering the total of 675 patients with a confirmed diagnosis of retinoblastoma, the male/female ratio was 1.3, ranging from 0.5 to 3.5 in the different local PBCRs. In 5 PBCRs, there were only male cases (Table 4).

**Table 4:** Quality indicators of Population-Based Cancer Register (PBCR) for cases of retinoblastoma in children and adolescents aged 0 to 19 years from the year 2000, National Cancer Institute, Ministry of Health, Brazil.

REGION	PBCR	Reference period	RTB cases (*n*)	RTB cases + malignant neoplasm (*n*)	Diagnostic method		M/F ratio
					DC^a^, *n* (%)	NR^b^, *n* (%)	
North	Manaus	2000–2013	23	25		2	1.88
	Belém e Ananindêua	2000–2017	27	28	1		1.25
	Rondônia	2015–2017	2	2			1.00
	Palmas	2000–2013	1	1	1		*
	Roraima	2003–2010	4	4		2	*
	**Total**	** **	**57**	**60**	** **	** **	** **
Northeast	Alagoas	2010–2011	7	7			0.75
	Aracaju	2000–2014	4	4			3.00
	Fortaleza	2000–2013	25	28	3		1.50
	João Pessoa	2000–2016	8	8			1.67
	Natal	2000–2008	18	19	1		3.50
	Recife	2000–2016	27	27			3.50
	Salvador	2000–2005	23	23		1	1.09
	Teresina	2000–2016	4	4			*
	**Total**	** **	**116**	**120**	** **	** **	** **
Midwest	Campo Grande	2008–2012	3	3			0.50
	Cuiabá/Várzea Grande	2000–2016	7	7			1.33
	Distrito Federal	2000–2014	28	30	1	2	0.75
	Goiânia	2000–2013	27	27		6	0.59
	Mato Grosso (inland)	2001–2016	9	10	1		1.25
	**Total**	** **	**75**	**77**	** **	** **	** **
Southeast	Angra dos Reis	2007–2016	3	3			0.50
	Belo Horizonte	2000–2017	39	40	1		1.60
	Campinas	2002–2016	8	9	1	1	0.60
	Barretos DRS	2000–2018	9	9		1	3.50
	Jahu	2000–2018	1	1			*
	Metropolitan área, ES^#^	2000–2012	7	8	2		1.33
	São Paulo	2000–2015	324	326			1.17
	**Total**	** **	**391**	**396**	** **	** **	** **
South	Curitiba	2000–2016	20	20			1.00
	Florianópolis	2008–2016	1	1			*
	Porto Alegre	2000–2012	16	17	1	3	1.67
	**Total**	** **	**37**	**38**	** **	** **	** **
**Total**	** **	** **	**675**	**691**	**13 (1.9)**	**18 (2.6)**	**1** **.** **30**

^a^ DC – number and percentage of cases whose only means of diagnosis was the death certificate, for a total of 691 cases.

^b^ NR – not reported, for a total of 691 cases.

* PBCR in which there were only cases in males.

^#^ ES = Espírito Santo state (cities of): Cariacica, Guarapari, Serra, Viana, Vila Velha, Vitória and Fundão.

**Figure 3:**
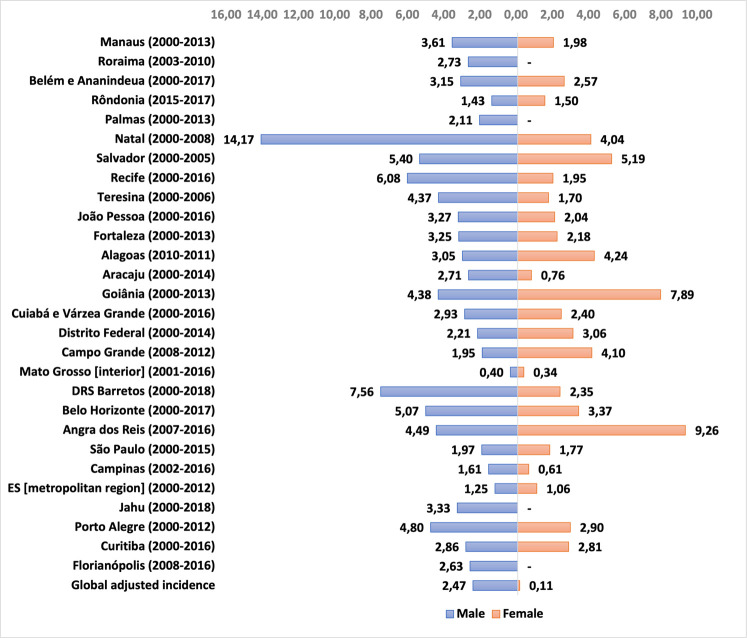
Retinoblastoma incidence rate per 1 million children and adolescents (0 to 19 years old), adjusted for age, according to sex and PBCR, in their respective reference periods.

The age-adjusted incidence rates of retinoblastoma, from 0 to 19 years, according to sex, in the different PBCR, in their respective reference periods, show a slight male predominance in most places, ranging from 0.40 within the state of Mato Grosso do Sul to 14.17 in the city of Natal, while in females the variation was from 0 in the cities of Florianópolis, Jahu and Palmas to 9.26 in the town of Angra dos Reis (Figure 3 and Supplementary Table S2).

**Figure 4:**
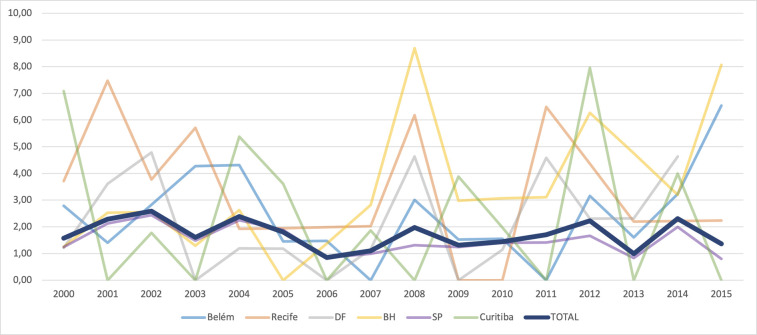
Time evolution of retinoblastoma incidences in six municipalities in the five Brazilian regions and the total combined incidence, from 2000 to 2015.

**Figure 5:**
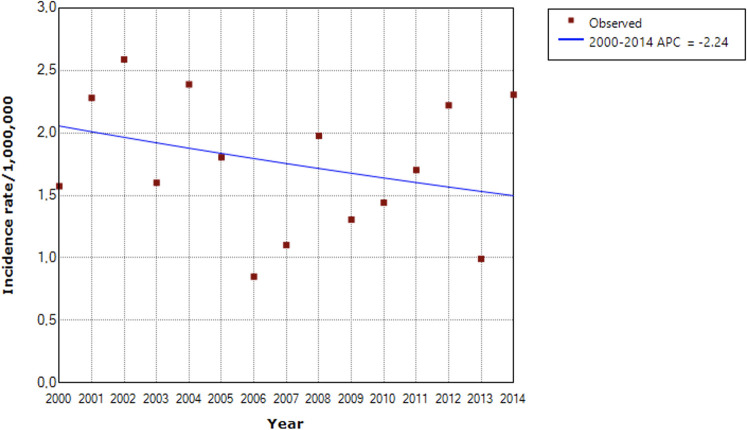
Time series from 2000 to 2014 demonstrating stationarity (stable trend) of the combined incidence of retinoblastoma in children and adolescents aged 0 to 19 years in six Brazilian municipalities.

**Figure 6:**
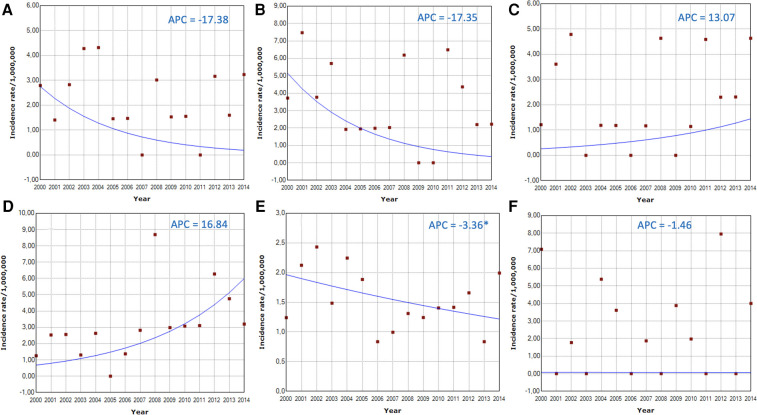
Time series from 2000 to 2014 demonstrating the trend in the incidence of retinoblastoma in children and adolescents aged 0 to 19 years in six Brazilian municipalities. (**A**) city of Belém and Ananindêua; (**B**) city of Recife; (**C**) city of Brasília (Federal District); (**D**) city of Belo Horizonte; (**E**) city of São Paulo; (**F**) city of Curitiba. Only the city of São Paulo showed a significantly downward trend (APC = −3.36%). The others and the combined incidence showed stationarity (stable trend).

The graph of the temporal evolution of retinoblastoma incidences in the six municipalities of the five Brazilian regions that had complete data from 2000 to 2014 shows large annual fluctuations. However, the total combined incidence has remained stable (Figure 4). The autoregressive analysis by inflection points (“joinpoints”) showed that the best model in all cities and the combined analysis was the one that did not assign any inflection points (“joinpoints” = 0). Thus, the APCs were identical to the AAPCs, and the final models showed stationarity (stable trend) in all cities, except for São Paulo, which showed a significant annual reduction of 3.4% (95%CI: −6.2%; −0.4%) (Table 5 and Figure 5). We show the regression lines and APCs of all regions in Figure 6. Complementary data from the trend analysis can be found in Supplementary Tables S3.

**Table 5:** Retinoblastoma mortality trends between 0 and 19 years of age, in six Brazilian cities, from 2000 to 2014, National Cancer Institute, Ministry of Health, Brazil.

PBCR	APC (%)	IC (95%)		*p*-valor	Trend
		LL	UL		
Belém/Ananindêua	−17.4	−42.6	18.9	0.278	Stationary
Recife	−17.3	−46.7	28.1	0.365	Stationary
Brasília (DF)	13.1	−21.2	62.2	0.475	Stationary
Belo Horizonte	16.8	−10.0	51.7	0.221	Stationary
São Paulo	−3.4*	−6.2	−0.4	0.028	**Downward**
Curitiba	−1.5	−29.7	38.2	0.927	Stationary
**Total**	**−2** **.** **2**	**−5** **.** **0**	**0** **.** **6**	**0** **.** **111**	**Stationary**

PBCR, population-based cancer registry; APC, annual percent change; CI, confidence interval; LL, lower limit; UL, upper limit; DF, Federal district.

* Negative APC, significantly different from zero at a significance level of 0.05, indicating a downward trend.

## Discussion

This is the first population-based study on the incidence of retinoblastoma in Brazil. Crude incidences varied considerably among the different local PBCRs and different Brazilian regions. Still, they remained stable over the years in most of the cities studied, except for the city of São Paulo, where it showed a significant downward trend. More than half of the cases occurred in the Southeast region of the country; however, the Northeast region had the highest crude incidence rates in children under five years of age and the highest ASIR, with values that were similar to those reported in developed countries, such as the United States, Canada, Finland, Sweden, and the United Kingdom. The adjusted incidence rates of the other Brazilian regions were lower than those of these countries. As expected, the highest incidence occurred in the age group from 0 to 4 years of age, with a slight predominance in males; however, about 10% of cases were diagnosed after five years of age.

Calculating the incidence of rare diseases with an early presentation, such as retinoblastoma, can be challenging. The estimation of the incidence of retinoblastoma may be affected by methodological issues. Due to the low number of cases per year, incidence rates can fluctuate and show random patterns, particularly in small populations. Another difficulty is defining the population at risk based on the child's age at diagnosis. Three different methods are reported in incidence studies: (1) the number of new cases in a given period per million children living in the same period; (2) the number of new cases in a given period by the number of live births in the same period; (3) the number of new cases in children born each year accumulated over time by the total number of live births in the same year (birth cohort analysis).

There is no consensus on the best way to perform this calculation. Seregard et al. (2004) and Li et al. (2016) (21, 26) compared the three methods and suggested that the birth cohort calculation produces the most accurate estimates, as they consider the real population at risk of developing the disease. As retinoblastoma is a developmental tumor and is rarely present at birth, live births or children existing at a particular location and period do not adequately represent the population at risk of developing the disease. Using them in the denominator could bias tumor incidence results, especially in countries with high infant mortality rates.

In the present study, we chose to calculate the incidence of retinoblastoma per one million children because this is the international standard recommended by the IARC for the World Cancer Incidence Report – Cancer Incidence in Five Continents (CIFC) (31) – based on the PBCR, enabling comparison with most international studies on cancer incidence (5, 13, 16). In countries with continental dimensions, such as Brazil, an additional problem for estimating the incidence of cancer is the limited coverage of existing PBCR that, although present in most of the main Brazilian capitals, do not cover the entire territoriality of the country, leading to probable underreporting of cases. Another challenge is to accurately define the coverage area of each local PBCR to determine the populations at risk associated with each one.

Indeed, in this study, except for the northeast region, in most local PBCR, we found lower incidences of retinoblastoma than reported in high-income countries (26). We believe this fact may be related to the difficulties pointed out in obtaining accurate estimates, added to the probable underreporting. We found that the number of new cases of retinoblastoma in the hospital-based registry of the INCA – Hospital Cancer Registry (RHC) – in the same period (2000–2018) is more than quadruple (*n* = 2821) of the total cases reported in the PBCR, evidencing the limited coverage of the population-based registry. However, the RHC cannot be used to calculate the incidence because despite involving more than 100 institutions that assist children and adolescents with retinoblastoma, it is not possible to guarantee that the number of cases registered in these institutions corresponds to all cases in Brazil, since other institutions that are not part of the RHC can receive patients with retinoblastoma. Therefore, it is incorrect to calculate the incidence using the entire population of children and adolescents in Brazil in the denominator, and it is impossible to specify the population at risk of presenting retinoblastoma. In most countries, the incidence of retinoblastoma assumes higher rates, such as those described in children under five years of age in Canada (11.6/1,000,000), the United Kingdom (10.0/1,000,000), the United States (10, 9/1,000,000) and India (9.6/1,000,000) (11, 16, 23, 28). As the Brazilian population is highly mixed due to the country's colonization process, we did not find in the literature genetic reasons that justify lower incidence rates of retinoblastoma in Brazil when compared to European countries.

On the other hand, our results are very consistent with a previous report published by the INCA in 2016 (7), including 24 local PBCRs. The medians of all local crude incidences per one million children were 7.13 (1–4 years), 0.00 (5–9 years, 10–14 years and 15–19 years), 3.35 (0–14 years), and 2.66 (0–19 years), similar to the pooled crude incidences of the 28 PBCRs included in the present study. The medians of the adjusted incidence were 3.98 (0–14 years) and 3.26 (0–19 years), higher than the pooled adjusted incidences we found [2.23 (0–14 years) and 2.24 (0–19 years)]. Some local incidences were lower or higher than those in the present study. Still, most of them were very similar to our findings, including the highest incidence previously exhibited in Natal, which was 22.34 in the previous report compared to 27.94 in the present study. These results corroborate the consistency of our estimates.

We found the highest gross and adjusted incidences in the northeast region of Brazil, similar to incidences reported in high-income countries (11). High incidence rates of retinoblastoma have been reported in low-income countries, suggesting that environmental factors related to poverty could account for the increased risk of retinal cell mutagenesis (30). Researchers have linked the increased risk of sporadic non-germ retinoblastoma to paternal work activities in the field with exposure to pesticides and a maternal diet low in folate, lutein, and zeaxanthin found in fruits and vegetables (34, 35). These nutrients participate in DNA synthesis, its methylation changes, and the formation of retinal cells. The northeast Brazilian region has essential agricultural activity and large pockets of poverty, which may be associated with pesticide exposure and poor eating habits, corroborating these hypotheses.

The assessment of the temporal trend of incidence of rare diseases such as retinoblastoma is also affected by random variation and significant annual fluctuation. In our study, an additional difficulty was the large difference between the reference periods of each local PBCR, which ranged from two years in some PBCRs to more than 18 years in others. We selected PBCR from capitals in each region that had data from at least 15 years from 2000 onwards and found a trend towards stability in all regions, except for the city of São Paulo, in the southeast region, the richest in the country, which presented significant annual reduction of 3.4% per year. It is difficult to explain this decreasing trend, but a hypothesis to be tested would be that there has been an improvement in care in the other regions, reducing the number of patients referred for treatment in the city of São Paulo. Our sample's representative cities of the North and Northeast regions also showed negative variations. In contrast, those of the Center-West regions and the city of Belo Horizonte – a large capital in the Southeast region – showed positive variations. Still, none of these variations reached statistical significance, being therefore considered to be of stationary trend. Nunmmi et al. (24) showed an increasing trend in the incidence of retinoblastoma in Finland when cases with a positive family history were included and a stable trend when these familial cases were excluded. The increasing incidence in familial cases probably reflects the current low mortality rate due to better treatment methods and, thus, greater inheritance of RB1 gene mutations associated with the familial form of the disease. The trend of increasing incidence evidenced in European countries such as Great Britain (23) and the Netherlands (36) can be attributed to improvements in diagnosis, registration, and investigation of cases. In our study, it was impossible to differentiate between cases with positive family inheritance due to the lack of this information in the PBCR.

The quality of the Information in a cancer registry depends on the accuracy of documents collected from sources. The evaluation of the diagnostic method of registered cases can reflect the degree of accuracy of the data and is one of the quality indicators of the PBCR. In the present study, the percentage of cases diagnosed only by the death certificate (DC) was very low (1.9%), which indicates good data quality. A high percentage of cases diagnosed with DC is considered a negative indicator, as the date of death is defined as the date of diagnosis of the tumor, which distorts the calculation of incidence rates. Another quality indicator in the present study, the incidence ratio between males and females, showed little difference between the sexes, consistent with the world literature (10).

Efforts to improve the detection, report, and registration of cases of retinoblastoma are crucial. The Brazilian Ministry of Health, the Brazilian Society of Pediatrics, and the Brazilian Society of Pediatric Ophthalmology are responsible for numerous documents and joint actions for the awareness of professionals and the population about the main signs, symptoms, and diagnostic and therapeutic methods of retinoblastoma to favor the early diagnosis of the tumor (37–41). They highlight the importance of the red reflex test and its performance requirement in all newborns, a procedure long suggested by the American Academy of Pediatrics for early screening of eye diseases (42).

Early detection of retinoblastoma can dramatically impact the prognosis of the disease and avoidance of childhood blindness. Strategies to improve the detection include training and standardizing the performance of the red reflex test by pediatricians. The mandatory test performance in newborns in all public and private maternity hospitals in the country represented a major advance in the screening for eye diseases. Despite being part of the neonatal care protocol in most Brazilian states, some still do not have legislation for performing the test. According to the Ministry of Health document “Childhood Eye Health Care Guidelines” (43), the red reflex test must be done within 72 h of birth and repeated at least three times a year for the first three years of life. In the presence of any abnormality, the child should be referred for consultation with a child ophthalmologist. Expanding legislation to all Brazilian states can guarantee the performance of the red reflex eye by the Unified Health System (SUS) and the National Supplementary Health Agency, as well as the referral of the neonate for diagnosis and management in a specialized unit once any alteration is detected. Another important point is parental education during routine pediatric consultation regarding the main clinical signs of the disease, such as leukocoria, strabismus, and proptosis, which the family often detects in photographs. Particular attention should be given to those families with consanguineous marriages and positive inheritances. The family member should be guided to immediately notify any of these signs to their pediatrician, who will be responsible for making the referral to the ophthalmologist. It is also important to emphasize the frequency of consultations with the ophthalmologist indicated by various medical societies of Ophthalmology and Pediatrics (44), which includes: red reflex test, blink assessment, and pupillary reflex in the newborn; second screening with the same tests between 6 and 12 months, checking for eye alignment and eye movements; photographic evaluation of the eyes between 12 and 36 months; assessment of visual acuity and eye alignment between 3 and 5 years; and annual visual acuity and eye alignment at 5 years or older. Finally, continuous public health programs in daycare centers and schools with eye screening services for children up to 5 years of age can also help in the early detection of the tumor.

Some limitations can be pointed out in the present study. The accuracy of the estimated incidence rates and their temporal evolution may be compromised for several reasons previously mentioned: rarity of the disease, probable underreporting of cases in areas not covered by the PBCR, difficulties in determining the populations at risk in each PBCR, lack of consensus on the best method of estimation and great variability in the reference periods of the different local PBCRs. The reported incidences, generally lower than those reported in high-income countries, suggest information and/or specification biases. The incidence estimated in the present study should be interpreted in light of these limitations. However, this is the first population-based study on specific incidence rates of retinoblastoma in Brazil, with the calculation of combined incidences to obtain the best and most up-to-date estimates possible at the national level, which may provide a better panel than the currently available. The last INCA report on cancer in children and adolescents published in 2016 includes reference periods up to a maximum of 2012 and does not present estimates of combined incidences, only the median of the incidences of the different local PBCR.

In conclusion, despite the good quality indicators related to retinoblastoma evidenced in the PBCR, the lower incidences in most Brazilian regions compared to the incidences reported in several countries around the world suggest underreporting cases. Let's consider the probable underreporting of cases in the poorest regions, where the incidences were higher. The rates in these places may be even higher than those estimated in this study. The results suggest that although we have advanced a lot in the registration of cancer cases since the implementation of PBCR in Brazil, many challenges remain for an accurate estimation of rare tumors such as retinoblastoma at the national level. Ongoing public health campaigns for the awareness of pediatricians and the general population about the disease can be an effective strategy to improve reporting. In addition, expanding the coverage of the PBCRs to more municipalities, providing continuous technical training to fill out the PBCR records correctly and avoid interruptions, and cross-referencing PBCR data with the Hospital Cancer Registry could improve the quality of the registries and allow a more accurate estimation of the national incidence of retinoblastoma. These estimates are essential for assessing the quality of a country's health system and serve as a reference to guide pediatric cancer care.

## Data Availability

The original contributions presented in the study are included in the article/**Supplementary Material**, further inquiries can be directed to the corresponding authors.
