# Supplementary material for: Incidence of retinoblastoma in children and adolescents in Brazil: A population-based study
Source: Front Pediatr. 2022 Nov 23;10:1048792. doi: 10.3389/fped.2022.1048792 (PMC9727227; doi:10.3389/fped.2022.1048792)
Supplement: Supplementary file 1 [file Table1.pdf]

Incidence of retinoblastoma in children and adolescents in Brazil: a population-based study

Annamaria Ciminelli Barbosa; Maria Clara de Magalhães-Barbosa; Jessica Pronestino de Lima Moreira; Giovanni Nicola Umberto Italiano Colombini and Arnaldo Prata-Barbosa.

TABLE S1 | Distribution of Retinoblastoma cases by five major geographic regions of Brazil. from 2000 to 2018. according to the Population-Based Cancer Registry (PBCR). from the National Cancer Institute. Ministry of Health. Brazil.

PBCR	Period of time	Age group (Years)				
		0 a 4	5 a 9	10 a 14	15 a 19	0 a 19
**North region**						
Manaus	2000-2013	21	2	0	0	23
Belém/Ananindeua	2000-2017	23	3	1	0	27
Rondônia	2015-2017	2	0	0	0	2
Palmas	2000-2013	1	0	0	0	1
Roraima	2003-2010	3	1	0	0	4
**Total**		**50**	**6**	**1**	**0**	**57**
**Northeast region**						
Alagoas	2010-2011	7	0	0	0	7
Aracaju	2000-2014	3	1	0	0	4
Fortaleza	2000-2013	22	3	0	0	25
Natal	2000-2008	16	2	0	0	18
João Pessoa	2000-2016	8	0	0	0	8
Recife	2000-2016	22	2	1	2	27
Salvador	2000-2005	21	2	0	0	23
Teresina	2000-2006	3	1	0	0	4
**Total**		**102**	**11**	**1**	**2**	**116**
**Midwest region**						
Campo Grande	2002-2012	3	0	0	0	3
Cuiabá e Várzea Grande	2000-2015	7	0	0	0	7
Distrito Federal	2000-2014	24	4	0	0	28
Goiânia	2000-2013	25	1	1	0	27
Mato Grosso Interior	2001-2016	6	3	0	0	9
**Total**		**65**	**8**	**1**	**0**	**74**
**Southeast region**						
Angra dos Reis	2007-2014	3	0	0	0	3
Belo Horizonte	2000-2016	37	2	0	0	39
Campinas	2010-2015	2	0	1	0	3
	2002-2005	5	0	0	0	5
Barretos (DRS)	2000-2018	8	1	0	0	9
Jahu	2000-2018	1	0	0	0	1
Metropolitan area. ES	2000 -2012	6	0	1	0	7
São Paulo	2000-2015	313	11	0	0	324
**Total**		**375**	**14**	**2**	**0**	**391**
**South region**						
Curitiba	2000-2015	18	1	0	1	20
Florianópolis	2008-2016	1	0	0	0	1
Porto Alegre	2000-2012	15	1	0	0	16
**Total**		**34**	**2**	**0**	**1**	**37**

*Espírito Santo state. cities: Cariacica. Guarapari. Serra. Viana. Vila Velha. Vitória. and Fundão.

frontiers

TABLE S2 | Incidence rates per 1 million children and adolescents for male and female retinoblastoma by Population-Based Cancer Registry (PBCR). National Cancer Institute. Ministry of Health. Brazil. by reference period and age group.

Region	PBCR	0 – 4 y/o		5 – 9 y/o		10 – 14 y/o		15 – 19 y/o		0 – 19 y/o			
		IR (crude)		IR (crude)		IR (crude)		IR (crude)		IR (crude)		ASIR	
		M	F	M	F	M	F	M	F	M	F	M	F
**North**	Manaus (2000-2013)	11.35	5.90	0.82	0.83	0.00	0.00	0.00	0.00	3.09	1.65	3.61	1.98
	Roraima (2003-2010)	9.93	0.00	4.96	0.00	0.00	0.00	0.00	0.00	2.59	0.00	4.22	0.00
	Belém / Ananindeua (2000-2017)	9.35	7.50	1.36	0.70	0.00	0.66	0.00	0.00	2.53	2.02	3.15	2.57
	Rondônia (2015-2017)	4.77	5.00	0.00	0.00	0.00	0.00	0.00	0.00	1.13	1.18	1.43	1.50
	Palmas (2000-2013)	0.00	0.00	0.00	0.00	0.00	0.00	0.00	0.00	0.00	0.00	2.11	0.00
	**Combined incidence**	**9.44**	**5.90**	**1.23**	**0.63**		**0.30**			**2.77**	**1.62**	**3.24**	**2.00**
**Northeast**	Natal (2000-2008)	44.52	10.69	3.24	3.33	0.00	0.00	0.00	0.00	11.0	3.15	14.17	4.04
	Salvador (2000-20005)	16.77	16.01	1.49	1.53	0.00	0.00	0.00	0.00	4.28	3.95	5.40	5.19
	Recife (2000-2016)	16.62	6.48	1.98	0.00	0.93	0.00	1.75	0.00	5.01	1.46	6.08	1.95
	Teresina (2000-2016)	11.40	0.00	3.81	0.00	0.00	0.00	0.00	0.00	3.63	0.00	4.37	1.64
	João Pessoa (2000-2016)	10.90	6.81	0.00	0.00	0.00	0.00	0.00	0.00	2.48	1.51	3.27	2.04
	Fortaleza (2000-2013)	10.27	6.10	0.69	1.41	0.00	0.00	0.00	0.00	2.51	1.68	3.25	2.18
	Alagoas (2010-2011)	10.17	14.12	0.00	0.00	0.00	0.00	0.00	0.00	2.34	3.19	3.05	4.24
	Aracaju (2000-2014)	9.02	0.00	0.00	3.05	0.00	0.00	0.00	0.00	2.13	0.71	2.71	0.76
	**Combined incidence**	**14.71**	**7.67**	**1.24**	**1.06**	**0.20**		**0.37**		**3.84**	**1.96**	**4.85**	**2.57**
**Midwest**	Goiânia (2000-2013)	13.56	25.06	0.00	1.50	1.39	0.00	0.00	0.00	3.50	5.98	4.38	7.89
	Cuiabá / Várzea Grande (2000-2016)	7.65	8.02	0.00	0.00	0.00	0.00	0.00	0.00	1.83	1.88	2.93	2.40
	Distrito Federal (2000-2014)	6.85	9.11	0.62	1.29	0.00	0.00	0.00	0.00	2.01	2.52	2.21	3.06
	Campo Grande (2008-2012)	6.49	20.48	0.00	0.00	0.00	0.00	0.00	0.00	1.53	4.75	1.95	6.14
	Mato Grosso (inland) (2001-2016)	0.85	0.89	0.56	0.29	0.00	0.00	0.00	0.00	0.34	0.29	0.40	0.34
	**Combined incidence**	**4.27**	**6.43**	**0.62**	**0.64**	**0.15**				**1.26**	**1.69**	**1.47**	**2.09**
**Southeast**	DRS Barretos (2000-2018)	22.22	7.82	3.56	0.00	0.00	0.00	0.00	0.00	6.13	1.77	7.56	2.35
	Belo Horizonte (2000-2017)	15.77	11.23	1.34	0.00	0.00	0.00	0.00	0.00	3.82	2.42	5.07	3.37
	Angra dos Reis (2007-2016)	14.97	30.86	0.00	0.00	0.00	0.00	0.00	0.00	3.43	7.02	4.49	9.26
	São Paulo (2000- 2015)	6.43	6.20	0.27	0.08	0.00	0.00	0.00	0.00	1.60	1.48	2.00	1.77
	Campinas (2002-2016)	5.36	0.75	0.00	0.00	0.00	1.72	0.00	0.00	1.26	2.16	1.61	0.61
	Metropolitan area/ES (Cariacica. Guarapari. Serra. Viana. Vila Velha. Vitória. and Fundão) (2000-2012)	4.50	3.52	0.00	0.00	1.07	0.00	0.00	0.00	1.35	0.83	1.59	1.06
	Jahu (2000-2018)	0.00	0.00	0.00	0.00	0.00	0.00	0.00	0.00	0.00	0.00	3.33	-
	**Combined incidence**	**6.98**	**5.18**	**0.34**	**0.14**	**0.03**	**0.03**			**1.80**	**1.52**	**2.19**	**1.60**
**South**	Porto Alegre (200-2012)	16.00	8.37	0.00	1.57	0.00	0.00	0.00	0.00	3.66	2.26	4.80	2.90
	Curitiba (2000-2016)	9.54	7.98	0.00	0.95	0.00	0.00	0.00	0.80	2.19	2.25	2.86	2.81
	Florianópolis (2008-2016)	0.00	0.00	0.00	0.00	0.00	0.00	0.00	0.00	1.97	0.00	-	-
	**Combined incidence**	**10.65**	**7.25**		**1.06**				**0.45**	**2.60**	**2.01**	**3.36**	**2.54**
**Total combined incidence**		**7.73**	**5.48**	**0.52**	**0.30**	**0.06**	**0.03**	**0.04**	**0.02**	**2.05**	**1.58**	**2.13**	**0.09**

IR (crude) – crude incidence rate; ASIR – adjusted incidence rate for world standard population.

TABLE S3 | Final model estimates of retinoblastoma incidence trends (ages 0-19 years) by joinpoints regression analysis in six Brazilian municipalities from 2000-2014, according to the Population-Based Cancer Register, National Cancer Institute, Ministry of Health, Brazil.

	Belém/Ananindêua	Recife	Brasília (DF)	Belo Horizonte	São Paulo	Curitiba	Conjugated data
**Nº Observations**	15	15	15	15	15	15	15
**Nº Parameters**	2	2	2	2	2	2	2
**DF**	13	13	13	13	13	13	13
**SSE**	312.337273	308.417839	573.662129	207.771239	32.366402	928.720441	45.496091
**MSE**	24.025944	23.724449	44.127856	15.982403	2.489723	71.440034	3.499699
**Auto Correlation**	-0.347994	-0.047813	-0.464997	-0.373212	-0.387617	-0.751123	-0.424701
**Segment**	0	0	0	0	0	0	0
**Joinpoints**	NA	NA	NA	NA	NA	NA	NA
**Intercept Estimate**	382.800869	382.645934	-246.992851	-311.718126	68.998771	26.833897	45.937613
**Intercept SE**	338.102743	406.835688	335.334305	242.957826	27.782215	314.253708	26.545136
**Intercept Test Statistic**	1.132203	0.940542	-0.736557	-1.283013	2.483559	0.085389	1.730547
**Intercept p-value**	0.278001	0.364089	0.474475	0.22189	0.027426	0.933253	0.107183
**Slope Estimate**	-0.190895	-0.190504	0.12282	0.155665	-0.034162	-0.014726	-0.022608
**Slope SE**	0.168509	0.202793	0.167044	0.120994	0.013845	0.156577	0.013228
**Slope Test Statistic**	-1.132847	-0.939402	0.735255	1.286547	-2.467382	-0.094048	-1.709132
**Slope p-value**	0.277740	0.364652	0.475241	0.220692	0.028274	0.926505	0.111174

DF – degree of freedom; MSE – mean squared error; SSE - sum of squared error; SE – standard error; NA – no joinpoint available
